# Pugilistic Science

**Published:** 1890-10-04

**Authors:** 


### The Hospital

A Weekly Journal of

**Science, Medicine, Nursing, and Philanthropy.**

*For Hospitals, Asylums, Medical Practitioners, Students, Nurses, Families, Parishes, Congregations, and the Charitable Public.*

Vol. IX.—No. 210.] [October 4, 1890.

### PUGILISTIC SCIENCE.

The pursuit of science, even of surgical science, is by no means always so dignified or so dull as the non-professional world is apt to suppose. There are still Fillgraves, who charge themselves with the honour of the profession, and treat the Thornes of less lofty bearing with all the scorn with which the Barchester physician was so abundantly endowed. But the Thornes get an innings sooner or later, and then they punish the bowling with undisguised goodwill. Sir Joseph Lister is not a Dr. Fillgrave, nor is Mr. Lawson Tait exactly a courteous Dr. Thorne; but Sir Joseph has certain of the Fillgrave peculiarities, and Mr. Tait is not without a liberal spice of Thorne's contempt for the mere conventional etiquette of the medical profession. Such as they are, the two have fallen out, or rather Mr. Tait has fallen foul of Sir Joseph Lister, and bestowed upon him some "slogging" strokes which cannot have been at all agreeable.

Why should these two surgeons quarrel, and what have they quarrelled about? The ostensible cause of their disagreement is a question of surgical science; the actual fact is, however, that the two men are so radically different in themselves that a quarrel was inevitable when fortune placed them both in the same field of work. Sir Joseph Lister represents the dignified surgeon and scientific man. He is nothing if not a man of dignity. He is also, if we mistake not, shy and reserved by nature. He can be and is kind, but not cordial. He impresses people with the idea that he is patronising them. Mr. Lawson Tait is in many respects the exact opposite of all this. He does not care for the outward and visible signs of dignity. He is a plain, blunt man—very blunt, and decidedly plain. He appears to hate pretence of all sorts, and he is one of those who think that men who are more ceremonious in manner than themselves are guilty of a foolish and reprehensible affectation. He is direct, not to say abrupt; hard down upon facts, and manifests a very marked contempt for those who like their facts wrapped up in a neat brown paper parcel of more or less plausible theory.

Such are the men. The cause which has ranged them on opposite sides is highly interesting from a scientific point of view, and of great practical importance in surgery. Sir Joseph Lister was the introducer of the antiseptic spray for surgical operations. His theory was, and is, that there are certain germs in the air which, when allowed to enter an open wound, set up putrefaction in it. Such being his theory, he logically set to work to devise a method of keeping the germs outside every open wound he had to deal with; and as surgical operations produce the most numerous and important of open wounds, it was imperative to find some mode of keeping away the germs of the atmosphere during the whole time of the performance of each operation. For this purpose an instrument was devised which produced a cone-like, medicated spray; the broad end of the cone being made to play on the part operated upon, and on the surrounding space for several feet. By this means, according to the theory, some of the germs of the air were forcibly driven away, whilst the others were killed. The result was, it was affirmed, that when the spray was used properly, and other antiseptic precautions, which we need not here detail, were employed, it was impossible that any otherwise healthy wound should putrefy or give rise to pus except what was of the most harmless kind.

The theory and practice thus briefly summarised were not only introduced by Sir Joseph Lister, but most strenuously advocated by him more than twenty years ago; and so successful was his advocacy that, after more or less opposition from his colleagues, notably the late Professor Spence and others, the whole surgical world accepted the Listerian theory and practice, and diligently carried it out in its minutest details. The theory and practice have maintained their ground up to the present time. But at the International Medical Congress, held at Berlin a few weeks ago, Sir Joseph Lister publicly declared his conviction that the "spray" was practically useless, and that he was ashamed he had ever introduced it.

In all this there seems to be no reason why either Mr. Lawson Tait or any other medical man should bear Sir Joseph Lister a grudge. Sir Joseph had introduced a detail of treatment, which, in course of time, he had found to be useless, and, thereupon, he frankly gave it up and confessed his error. Why, then, does Mr. Tait take the occasion of his rival's recantation for administering to him a very sarcastic and energetic scolding, after the manner of a big school-boy, who is full of delight because he has unexpectedly found one of his teachers in the wrong? Mr. Tait triumphs and scolds all in a breath, because, as he affirms, he has persistently declared the "spray" to be useless for a dozen years; and has done his utmost to prove the validity of his contention by performing hundreds of the most serious class of surgical operations without using it once. Those operations have been singularly successful; and taken in conjunction with similar operations performed by Dr. Granville Bantock and others, have been largely instrumental in convincing Sir Joseph Lister of the

141545

futility of the spray. Mr. Tait justifies his anger and his triumph on the ground that Sir Joseph has never once condescended to notice his arguments and facts during the whole dozen years in which he has been proclaiming them to the surgical world. Now, when he can no longer ignore either Mr. Tait or his facts, he professes to candidly recognise both.

What, then, is Mr. Tait's view of the germ theory? He admits the existence of germs; but denies that they have any influence on wounds unless the wounds are in some way unhealthy. In other words, while Sir Joseph Lister looks upon germs as the chief causes of deadly mischief in wounds, Mr. Lawson Tait says that if all the blood and other tissues which are separated from the living parts during an operation be carefully cleared away, the germs can do no harm. Sir Joseph Lister dreads germs, and germs only; Mr. Lawson Tait dreads dirt and exuded blood, and injured and separated portions of tissue which may be left lying in and around a wound, and these only.

What is the true science of the matter? Who is right, Sir Joseph Lister or Mr. Lawson Tait? Both of them certainly! Nay, we do not doubt that Sir Joseph Lister himself would agree with Mr. Tait that the germs of the atmosphere are likely to do little harm if a wound is kept absolutely clean; whilst if Mr. Tait were quite candid he would admit that there are certain atmospheric germs which are so injurious that it is impossible to take too much pains to deprive them of all possibility of multiplying, by keeping every portion of a wound as clean and sweet as care and pains can make it. It was not, perhaps, unnatural that Mr. Tait should feel injured by Sir Joseph Lister's indifference to his published facts and opinions. But he might have remembered that Sir Joseph himself had formerly endured a long period of like indifference at the hands of his seniors; that he had spent many years of the best part of his life in establishing his theory; and that he would have been more than human if he had given it up at the bidding of the first critic who cried stand and deliver, even though that critic was Mr. Lawson Tait. Besides, Mr. Tait might have called a little pride to his aid, and, if Sir Joseph Lister had ignored him, he might have retorted by ignoring Sir Joseph Lister. Neither Sir Joseph Lister nor any other single man constitutes the whole medical and surgical world. Mr. Tait is undoubtedly right in his practical conclusions about the spray; he is as certainly wrong in his affected contempt for the dangers of atmospheric germs; and he is most ungracious and undignified in his manner of dealing with an honoured man of science, who, when he finds he has been in error for twenty years has the courage and the self-denial to admit his error and to take prompt steps to undo it. We are glad to acknowledge Mr. Tait's capacity and success as a surgeon; but we cannot congratulate him upon the generosity of his conduct as a man of the world and as a member of the medical profession.

